# Recombinant SAG1 Antigen to Detect *Toxoplasma gondii* Specific Immunoglobulin G in Human Sera by ELISA Test

**Published:** 2010-06

**Authors:** N Jalallou, M Bandepour, H Khazan, A Haghighi, Sh Abdollahi, B Kazemi

**Affiliations:** 1 Dept. of Medical Parasitology and Mycology, School of Medicine, Shahid Beheshti University, M.C., Tehran, Iran; 2 Cellular and Molecular Biology Research Centers, Shahid Beheshti University, M.C., Tehran, Iran; 3 Dept. of Medical Microbiology, School of Medicine, Rafsanjan University, Rafsanjan, Iran

**Keywords:** *Toxoplasma gondii*, Recombinant SAG1, ELISA

## Abstract

**Background:**

Although some serological tests for the detection of *Toxoplasma gondii*-specific immunoglobulin are commercially available, better diagnostic tools are needed. The aim of present study was to evaluate the usefulness of the recombinant *Toxoplasma gondii* SAG1 antigen for the recognition of toxoplasmosis by ELISA.

**Methods:**

This study was conducted in Cellular and Molecular Biology Research Centers, Shahid Beheshti University, M.C., Tehran, Iran in 2008-2009. Surface antigen 1 (SAG1), a tachyzoite stage-specific protein, was subcloned into an expression vector and was subsequently transformed into BL21 (DE3) pLysS competent bacterial cells. After inducing expression of the recombinant antigen, the protein product was purified using Ni-affinity chromatography. The immunoreactivity of recombinant SAG1 (rSAG1) was analyzed by SDS-PAGE and western blotting. The reactivity of the rec-SAG1 protein was evaluated using an ELISA.

**Result:**

Sensitivity and specificity of the generated recombinant-ELISA (rec-ELISA) compared to a commercially available ELISA (com-ELISA) were 88.4% and 88%, respectively.

**Conclusion:**

Recombinant SAG1 produced in *E. coli* is a promising antigen that can be used in diagnostic assays for the detection of specific antibodies against *T. gondii*.

## Introduction

Toxoplasmosis is caused by an obligate intracellular protozoan parasite, *Toxoplasma gondii*, which is able to infect most mammals and birds (1). It is estimated that toxoplasmosis exists in a chronic asymptomatic form in 500 million to 1 billion people (2). In humans, *Toxoplasma gondii* is generally asymptomatic but in pregnant women can result in congenital infection with severe sequelae or late onset eye disease. *T. gondii* is also a frequent cause of encephalitis in severely immuno-suppressed patients with AIDS (3, 4). Additionally, toxoplasmosis is a serious complication following organ transplantation (5).

Diagnosis of *T. gondii* infection can be established in fetus and new-born infants by the isolation of *T. gondii* from blood or body fluids, by demonstration of the parasite in tissue, and by detection of specific nucleic acid sequences with DNA probes (6). Laboratory diagnosis of *Toxoplasma* infection is usually based on the detection of specific antibodies. The specificity and sensitivity of these methods depend primarily on the diagnostic antigens (7). Many serological tests used in the detection of *T. gondii*-specific immunoglobulin are commercially available, the majority of which use native parasite antigens prepared from tachyzoites grown in mice and/or *in vitro* tissue culture that contain various non-parasitic materials from the culture media and the eukaryotic host cells (8–10). The enzyme-linked immunosorbent assay (ELISA) is one of the easiest tests to perform. Due to the lack of a purified standardized antigen or a standard method for preparing the antigen, it is not surprising that some interassay variability exists (9).

The major advantages of using recombinant antigens in the diagnosis of *T. gondii* infections are as follows: (a) the antigen composition of the test is precisely known, (b) more than one defined antigen can be used, and (c) the method can be easily standardized. Therefore, the use of recombinant antigens would allow better standardization of the tests and would reduce the costs of production. These considerations are very important when, as often happens, only one serum sample is available for testing (11).

To develop a standardized antigen, recombinant SAG1 (previously named p30) was produced in bacterial cells and purified. This antigen is one of the principle proteins in tachyzoites, and because of its immunological structure, SAG1 is considered an important candidate for the development of effective diagnostic reagents or subunit vaccines that induce an immunodominant response (12). This antigen is suitable for use in diagnostic systems for detecting anti-SAG1-specific IgG and IgM antibodies. The recombinant SAG1 has no cross-reactivity with proteins from other microorganisms (13).

The aim of present study was to evaluate the usefulness of the recombinant *T. gondii* SAG1 antigen for the recognition of toxoplasmosis by ELISA.

## Materials and Methods

### Subcloning

SAG1 antigen (accession number EF140712) was cloned into the pQE30 vector (14) and then subcloned into the pET32a (code: PEC 018, NRGB) expression vector. The sequence of the insert was confirmed by PCR (pET32a primers: F 5'- AGG GGT TAT GCT AGT TAT TG -3' and R 5'- CTG CTA AAT TCG AAC GCC A -3'; Tox P30 primers: F 5'- GGT ACC ATG TTT CCG AAG GCA GTG -3' and R 5'- AAG CTT CGC ACA CAA GCT GCG AT-3') and by restriction analysis using Pst1 (Fermentas, Lithuania Cat).

### Gene expression

The recombinant plasmid was transformed into *Escherichia coli* BL21 (DE3) pLysS competent cells. A single colony was grown in LB medium (Merck Frankfurte, Germany,) containing 100 µg/ml ampicillin overnight at 37°C and then diluted 10-fold with fresh LB medium contain ampicillin. The plasmid promoter was induced with isopropyl-D-thiogalactopyranoside (IPTG) at a final concentration of 1 mM. The cells were incubated with vigorous shaking at 37°C for 7 h. The cells were harvested by centrifugation (10,000 rpm for 10 min). The expressed protein was confirmed by SDS-PAGE and western blot analysis.

### Protein purification

Purification was performed using Ni-affinity chromatography (Novagen, Madison, USA) according to the manufacturer's protocol with some modification. The cell pellet (extracted from 25 ml of LB medium) was resuspended in 4 ml of equilibration buffer (500 mM NaCl, 50 mM Tris-HCl, 0.5 M Urea) plus 1 mM PMSF (phenylmethanesulfonyl fluoride) and incubated overnight at 4°C. The suspension was then sonicated and centrifuged (10,000 rpm for 20 min at 4°C). The supernatant was collected and transferred to a Ni-NTA column containing 2 ml equilibrated resin. The column was incubated overnight at 4°C and was washed with 10 ml washing buffer (1 M NaCl, 50 mM Tris-HCl, 0.4 M urea). The bound recombinant protein was eluted with 2 ml elution buffer (500 mM NaCl, 50 mM Tris-HCl, 1 M imidazole, 0.4 M urea). The eluted fraction was dialyzed against PBS buffer (10 mM Na_2_HPO_4_, 150 mM NaCl) and the protein concentration was measured by BioPhotometer (Eppendorf, Hamburg, Germany).

### Western blot analysis

The immunoreactivity of recombinant His_6_-SAG1 was analyzed by SDS-PAGE with Coomassie brilliant blue (G 250) staining. The protein was subsequently transferred to a nitrocellulose membrane (Porablot, Düren, Germany) using a semi-dry blotting apparatus (Apelex, France). Human serum samples containing *T. gondii*-specific IgG antibodies or a His-tagged monoclonal antibody diluted in blocking solution (1:500 and 1:1000, respectively) were used as the primary antibodies. Rabbit anti-human IgG and goat anti-mouse IgG, diluted 1:000 and 1:5000 in blocking solution, respectively, were used as the secondary antibodies. The membrane-bound rSAG1 was detected with DAB (3, 3'-diaminobenzidine; Sigma, Munich, Germany).

### ELISA

Each well of flat-bottomed polystyrene microplates (Greiner Bio One, Frickenhausen, Germany) was coated with 100 µl purified recombinant protein at a concentration of 4 µg/ml in 50 mM carbonate-bicarbonate buffer (pH 9.6) overnight at 4°C. Wells were washed three times (PBS, pH 7.2, 0.05% Tween 20) and incubated with blocking solution (1% BSA and 0.05% Tween 20 in PBS) at room temperature for 2 h. After blocking, 100 µl of human sera, diluted 1:100 in blocking solution, was loaded into each well and incubated for 1.5 h at room temperature on an orbital shaker. After another round of washing, each well was incubated with 100 µl of polyclonal rabbit anti-human IgG horseradish peroxidase-conjugated secondary antibody (Dako Cytomation, Produktionsvej, Denmark) diluted 1:10,000 in blocking solution for 1 hour at room temperature on an orbital shaker and then washed as in the previous step. The OPD (Dako, Produktionsvej, Denmark) substrate (100 µl; 8 mg of OPD was dissolved in 12 ml of distilled water and 4 µl H_2_O_2_ prior to use) was added. After 10-min incubation at room temperature in the dark, 50 µl of 2 N sulfuric acid was used to stop the reaction. The absorbance at 492 nm was measured using an automated microplate reader (Sunrise-Tecan, Männedorf, Switzerland). The cut-off value was calculated as the mean level of negative sera plus two standard deviations.

A total of 173 sample sera (153 sample sera from patients with suspected *T. gondii* infection and 20 sera from patients with other diseases) were tested using a commercial ELISA kit (Euroimmun, Lübeck, Germany) prior to testing them using our ELISA. Using the results of com-ELISA, sera were classified as negative or positive for *T. gondii*.

## Results

**Fig. 1:**
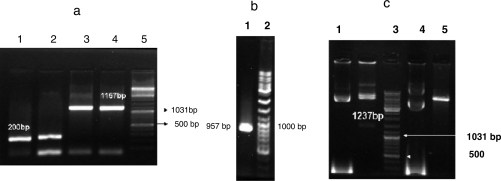
Confirmation of subcloning. A PCR was performed using pET32a (panel a) and ToxoP30 primers (panel b). The resulting amplicon was separated by electrophoresis on a 1.5% agarose gel. The reaction of confirmation enzyme is shown in panel c. Panel a: lanes 1-2, pET32a without the SAG1 insert (200 bp); lanes 3-4, pET32a containing the SAG1 insert (1157 bp); lane 5, DNA ladder. Panel b: lane 1, SAG1 gene (957 bp); lane 2, DNA ladder. Panel c: lane 1, uncut recombinant pET32a; lane 2, Pst1-digested recombinant pET32a (1237 bp); lane 3, DNA ladder; lane 4, uncut pET32a, Pst1 digested pET32a (without the 1237-bp band)

The SAG1 gene was subcloned into pET32a and the resulting recombinant plasmid was confirmed by PCR and sequencing methods (Fig. 1a, b and c).

The Kpn1 and Sal1 restriction sites used for the ligation of SAG1 were 132 and 186 bases downstream of thioredoxin, which consists of 327 nucleotides. The resulting SAG1-thioredoxin fusion product had a molecular weight that was about 16 kDa higher than native SAG1.

**Fig. 2:**
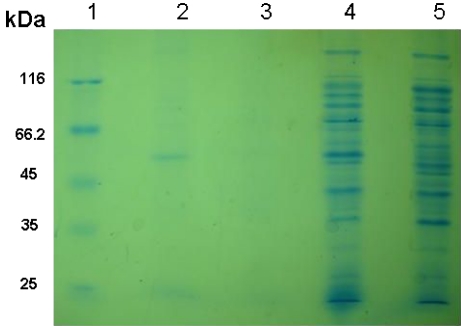
SDS-PAGE analysis of rSAG1 expression using 15% SDS-PAGE. Lane 1, molecular protein marker (Fermentas, Lithuania Cat). Lane 2, purified rSAG1 protein. Lane 3, blank. Lane 4, expression of SAG1 fusion protein at 52.4 kDa after 7 h of induction. Lane 5, induced control culture of cells lacking the SAG1 insert

The concentration of the purified protein that was measured by the BioPhotometer was 5 mg/ml. Before and after purification, a protein band was observed at 52.4 kDa on a 15% SDS-PAGE gel (Fig. 2). This band was absent in uninduced cultures and in an induced control culture of cells lacking the SAG1 insert.

**Fig. 3:**
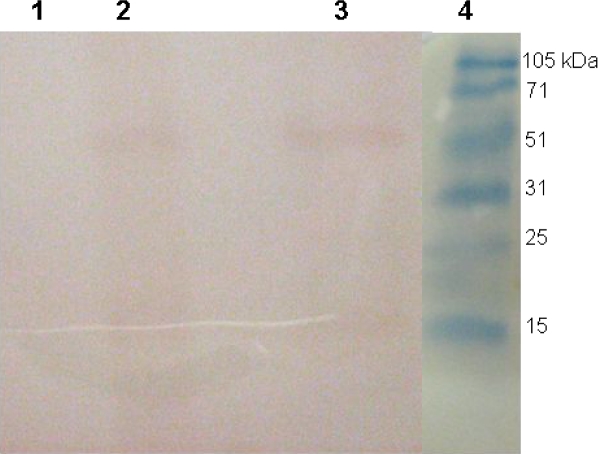
Western blot analysis of the rSAG1 protein using a monoclonal antibody targeting the His-tag. Lane 1, induced control culture of cells lacking the SAG1 insert Lane 2, expression of the SAG1 fusion protein at 52.4 kDa. Lane 3, purified rSAG1 protein. Lane 4, molecular protein marker

The immunoreactivity of the antigen before and after purification was confirmed by western blot analysis using a monoclonal antibody against His-tag in addition to the human serum samples, which were previously characterized for *T. gondii* specific IgG reactivity (Fig. 3).

**Fig. 4:**
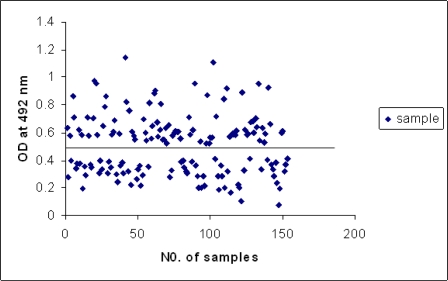
Profile of the antibody response in the rec-ELISA of human sera using the rSAG1 protein (cut-off value: 0.477)

The rSAG1 protein expressed in *E. coli* was evaluated by ELISA to assess whether it would be a useful antigen for serodiagnosis of toxoplasmosis. One hundred and seventy-three serum samples were tested by both the com-ELISA kit and the rec-ELISA. Twenty serum samples had IgG antibodies against other diseases (Table 1), although they appeared to be negative for *T. gondii*. The cut-off value was set equal to the average OD value of the negative population plus two standard deviations (Fig. 4).

**Table 1:** Diseases tested for cross-reactivity to rSAG1

Diseases	EBV	VZV	HSV	Rub	Measles	M.t.	H.p.	CMV
Number	4	2	2	2	2	3	3	2

EBV: Epstein-Barr virus; VZV: Varicella zoster virus; HSV: herpes-simplex virus; Rub: rubella; M.t: *Mycobacterium tuberculosis*; H.p: *Helicobacter pylori*; CMV: Cytomegalovirus

The result of the commercial and recombinant ELISAs are summarized in Table 2. The sensitivity and specificity of our assay using *T. gondii* recombinant protein antigens with respect to the com-ELISA were 88.4% and 88%, respectively.

**Table 2:** Comparison of com-ELISA and rec-ELISA

		com-ELISA		
		Positive	Negative	Total
	Positive	76	8	84
rec-ELISA	Negative	10	59	69
	Total	86	67	153

## Discussion

There have been several reports regarding the expression and purification of *T. gondii* antigens using various expression systems including *E. coli*, a mammalian cell expression system, or *Pichia pastoris* (15–18). Here we subcloned the DNA sequence of the *T. gondii* SAG1 protein into the pET32a T7 promoter-based expression vector. In this vector, the insert is expressed as a fusion protein with *E. coli* thioredoxin (trxA). This protein has been stably expressed at high levels in the pET system and is extremely soluble in the *E. coli* cytoplasm (19). In addition to its solubility, trxA is small (109 aa; 11,675 kDa), has inherent thermal stability, is localized on the cytoplasmic face of the adhesion zones between the inner and outer cell membranes and can be exploited for rapid purification (20). The rec-protein was purified using a simple purification method according to the manufacturer's protocol with some modification. The identity rec-protein was confirmed by western blotting, which showed the immunoreactivity of the specific protein. Thus, we successfully produced large quantities of highly purified protein in our laboratory.

Routine diagnosis of *T. gondii* infection relies primarily on serological recognition. A precise distinction between the acute and latent forms may be difficult since IgM antibodies, which are considered to be specific markers of early infection, may be present in sera for many years (21). An alternate, more reliable diagnostic test is needed.

Here, we evaluated the development of an ELISA technique using rSAG1 and its usefulness for serodiagnosis of toxoplasmosis in humans.

A robust immunological response to the surface antigen SAG1 is associated with chronic *Toxoplasma* invasion (22). In our study, rSAG1 was recognized in more than 88% of positive sera from IgM-negative patients.

Buffolano et al. (23) previously reported that SAG1 reacted with 75% of the sera from congenitally infected infants. In a survey published by Aubert et al. (9), rSAG1 rec-ELISA could recognize 83% of IgG antibodies against toxoplasmosis in chronically infected individuals, which is consistent with our results.

Pietkiewiez et al. used sera from patients with indicative infections acquired in the distant past (chronic toxoplasmosis) and showed that increasing the level of antibody titers increased the ability of rSAG1 to recognize positive sera (from 70% to 100%) (24). These results are within the range of our findings.

In agreement with the results obtained by Velmurugan et al. (25), the specificity and sensitivity of rSAG1 were 88.4% and 83.3% in goat sera, respectively. These percentages are very similar to our data.

In contrast, Nigro et al. (26) showed low or no reactivity against rSAG1. It is possible that they used a truncated gene and a purification method that resulted in incorrect folding of the recombinant protein.

We did not observe any cross-reactivity with other vertically transmittable diseases (Cytomegalovirus, Herpes simplex virus, Rubella, Varicella zoster virus), mycobacterium tuberculosis, Helicobacter pylori, Epstein bar virus, or measles using the rSAG1 protein. This finding verifies results of Harning et al. (13).

In conclusion, recombinant SAG1 produced in *E. coli* is a promising antigen that can be used in diagnostic assays for the detection of specific antibodies against *T. gondii*. Assays based on recombinant proteins are easier to standardize and are more reproducible because only a single protein with a few immunodominant epitopes is used (13).
